# A case of thyroid storm caused by Graves’ disease misdiagnosed as panic attack due to panic disorder

**DOI:** 10.1186/s13030-021-00211-4

**Published:** 2021-05-27

**Authors:** Manabu Yasuda, Jun Kumakura, Kiyonori Oka, Kazuhito Fukuda

**Affiliations:** 1 Department of Psychiatry, Jichi Medical University, Yakushiji, Shimotsuke-shi, Tochigi 329-0498 Japan; 2 Division of Endocrinology and Metabolism, Department of Internal Medicine, Jichi Medical University, School of Medicine, Yakushiji, Shimotsuke-shi, Tochigi 329-0498 Japan

**Keywords:** Case report, Graves’ disease, Panic attack, Thyroid hormone, Thyroid storm

## Abstract

**Background:**

Graves’ disease is characterized by hyperthyroidism and its symptoms often overlap with those of panic disorder, which may make it difficult to distinguish between the two conditions. In this report, we describe how proper diagnosis of thyroid disease in patients with mental illness can lead to appropriate treatment.

**Case presentation:**

We encountered a 34-year-old woman in whom thyroid crisis from Graves’ disease was misdiagnosed as panic attack. The patient was being managed as a case of panic disorder and bipolar disorder in a psychiatric outpatient setting. About 6 months before presentation she had lost about 16 kg in weight, and a month before presentation she developed several unpleasant symptoms as her condition worsened. Several weeks before she had had severe palpitations, tachycardia, and discomfort in her throat. She became unable to eat solids, ate only yogurt and gelatin, and felt it difficult to take psychiatric drugs.

She visited our emergency outpatient department on a Sunday morning, presenting with nausea, severe tachycardia, fever, and restlessness with anxiety. We treated her as panic disorder with fever, but noted proptosis and considered the possibility of Graves’ disease. Thyroid function tests were performed even though data from her clinic was not available because it was a weekend.

Because there was no improvement in her condition after her first visit, she returned to our hospital early the next morning. We had misdiagnosed her as having severe panic attacks due to panic disorder, and after a diazepam injection had allowed her to go home.

Later that day, the thyroid function test results became available, and her symptoms and the results strongly indicated a thyroid storm. The endocrinology department was consulted immediately, and she was referred for hospitalization the next day. During hospitalization, she was treated with steroid and radioactive iodine therapy and was discharged from hospital in 3 weeks.

**Conclusion:**

Psychiatrists and doctors engaged in psychosomatic medicine need to consider the possibility of severe hyperthyroidism as a differential diagnosis of panic disorder.

## Background

Graves’ disease is a form of hyperthyroidism characterized by the presence of thyroid-stimulating hormone receptor (TSHR) autoantibodies, which mimic the effects of thyroid stimulating hormone (TSH) [1].

Treatment of Graves’ disease involves the use of methimazole or propylthiouracil, radioactive iodine therapy, or surgery. The prognosis is good with appropriate treatment, but outcome might be fatal if thyroid storm occurs and is left unchecked. In such cases, mortality is over 20% [1].

The symptoms of Graves’ disease are varied and include increased heart rate, elevated blood pressure, arrhythmia, profuse sweating, hot flashes, tremors, nervousness, and feelings of anxiety, all of which are also symptoms of panic disorder [2]. Therefore, the differential diagnosis of Graves’ disease and panic disorder can be difficult.

However, several studies about the relation between Graves’ disease and mental disorder, including panic disorder, have been conducted in Japan and abroad [3–6].

In a clinical survey of psychiatric disorders that included 93 cases (9 male and 72 female) with thyroid disease [3], those with comorbid Graves ‘disease and panic disorder comprised 36.1%, suggesting an association between Graves’ disease and panic disorder.

One of the Japanese studies [4] suggested that mental disorders, depression and anxiety often merge with Graves’ disease. The other studies from abroad [5] showed that mental disorders improve after thyroid function is normalized, but it is also known that mental symptoms remain in many patients after thyroid function is normalized [6].

We encountered a 34-year-old female patient in whom thyroid storm due to Graves’ disease was misdiagnosed as panic attack. The patient has given written informed consent for her case to be published in a form that does not violate her privacy and that protects her personal information.

## Case presentation

The patient was a 34-year-old woman who had previously been active and had studied abroad. She underwent surgical removal of a neck polyp at around the age of 12 years and thereafter noticed nausea and problems in her throat.

At the age of 16, she had depressive mood, anxiety, and palpitations and visited a psychiatric outpatient clinic in Tokyo, where she was diagnosed and treated as panic disorder and depression,

She has visited our hospital for 10 years for the treatment of panic disorder and bipolar disorder due to the appearance of manic state. She was treated with trazodone 50 mg/day, escitalopram 10 mg /day, and diazepam 2 mg /day, and there was no evidence of Graves’ disease such as persistent tachycardia.

At age 34 years, she developed a mild fever in April. A month later, she felt compression in her chest. Depression, vomiting, palpitations, and tachycardia ensued as her condition worsened.

Six weeks later, she had intense palpitations, tachycardia (140/min), had difficulty with things getting stuck in her throat when eating and brushing her teeth. She was able to consume only yogurt and gelatin. She also complained of weight loss, from 84 kg to 68 kg over a 6-month period, though she felt herself obese and was consciously losing weight.

Two months later, on a Sunday morning, she visited our emergency outpatient department with severe nausea, blood pressure 182/111 mmHg, heart rate 153/min, body temperature 37.7 C, and restlessness with anxiety. At her medical examination, panic disorder was suspected because of her medical history, but she had a fever so we examined the pharynx and collected blood to assess her physical condition.

Although there was no sign of infection in the pharyngeal findings or blood examination, they revealed anemia and dehydration, and she was given an iron preparation by oral administration.

Proptosis was noted in her examination and the possibility of Graves’ disease was considered, so thyroid function tests were performed even though it was a weekend.

She was allowed to go home, but her condition did not improve and she returned to the hospital at 4 am on Monday. She complained of feelings of dysphoria with severe palpitations, tachycardia, and nausea and was diagnosed as having panic attacks due to preexisting panic disorder.

We administered diazepam 10 mg/ampoule, and she became calm and was allowed to go home. At that time, the thyroid function test results were unavailable until Monday morning at the earliest, so the test results were unknown.

On Monday morning, her blood test results revealed the following: TSH < 0.02 μU/mL (standard ranges:0.45–3.33), free triiodothyronine > 25.00 pg/mL(2.11–3.51), free thyroxine > 8.00 ng/dL (0.84–1.44), total-triiodothyronine 10.86 ng/mL (0.84–1.40), and total thyroxine 55.50 μg/dL (5.15–10.43), thus thyroid crisis was suspected.

Because her condition could be expected to worsen, the attending physician immediately consulted with the endocrinology department and the patient was referred to them for further specialist management.

We evaluated her symptoms with the Burch Wartofsky scale on hospitalization. Of her 50 total points, severe nausea was 10, heart rate 25, body temperature 5, and restlessness with anxiety 10, so thyroid storm was strongly suspected [7, 8].

After hospitalization in the department of internal medicine, serum thyroid hormone and TSH receptor antibody levels were found to be high at 36.8 IU/L, so the patient was diagnosed as having thyroid crisis due to Graves’ disease. Treatment was started with dexamethasone injection 6.6 mg/day, methimazole 80 mg/day, and potassium iodide 200 mg/day. Propranolol 60 mg/day was added when the patient requested radioactive iodine therapy because of continued fever and tachycardia.

She was discharged from hospital in 3 weeks, after radioactive iodine therapy. Even after the isotope treatment, methimazole was not effective and was stopped. Propylthiouracil treatment was started because the thyroid profile showed no tendency to increase thyroid hormone. At 1 year after discharge she needed no psychiatric drugs, so consideration was given to termination of treatment. At 3 years after discharge, her depression recurred and she began taking antidepressants, though not as much as before Graves’ disease was diagnosed.

## Discussion and conclusions

There have been several reports on the distinction between panic disorder and thyroid storms and the difficulty in distinguishing between them given at conferences; however, case reports are exceptionally rare [9], so we felt our clinical experience would be worth reporting. It is notable that after treatment for Graves’ disease, psychiatric treatment with antidepressants needed to be continued for this patient, but not as much as before the Graves diagnosis.

We would like to make some clinical reflections. First, we were trapped by preconceived notions of her illness because she had already been diagnosed as panic disorder. Once a disease has been diagnosed, it is difficult to remove such preconceptions. The similar clinical symptoms of panic disorder and Graves’ disease, such as palpitations, nausea, restlessness, and weight loss, including the fact that she was losing weight to improve obesity, led us to this misunderstanding.

The tachycardia and restlessness of panic disorder is generally ameliorated by the administration of anxiolytics [2, 10]. In contrast, the tachycardia of Graves’ disease is not. More complete clinical evidence would help in more quickly making a correct diagnosis.

Secondly, this incident happened when the doctors were on weekend duty. In Japanese hospitals, medical resources effectively used on weekdays are often not available weekends, and fewer medical staff are available than on weekdays.

In this case, blood was collected on Sunday morning, but thyroid hormone results were not known until Monday, resulting in the delay of the correct diagnosis. It is difficult to make this diagnosis using only evaluations other than those that include the full complement of blood related items.

Finally, on-duty doctors often feel a responsibility to finish their shift without any problems. They are often required to make decisions on their own, without the specialist assistance that would be available on weekends and holidays. When doing a medical examination, the doctor can easily overlook important clinical findings and thus make critical mistakes, especially late at night or early in the morning when a doctor can be exhausted, which also increases the risk of making a misjudgment.

In this case, the author responded as the doctor on duty. In fact, early that morning I was quite exhausted, so may have hurried my conclusions and given in to the preconceived notion that she was again suffering a panic attack, without giving sufficient consideration to the possibility that she was suffering from another disease, such as a thyroid storm. Fortunately, a laboratory technician confirmed the thyroid hormone item on Monday morning and immediately reported the results to the outpatient psychiatrist, who was able to deal with it and save her life. Death could have occurred if the report had been further delayed.

The symptoms of panic disorder and Graves’ disease often overlap, which makes it difficult to distinguish between the two conditions. There is a high risk of misdiagnosis, especially on weekend or holiday shifts where medical resources are scarce and when fatigue and exhaustion set in.

Psychiatrists and doctors engaged in psychosomatic medicine should consider the possibility of severe hyperthyroidism as a differential diagnosis of panic disorder with severe panic attacks, testing thyroid hormone items and confirming the levels as soon as possible.

## Data Availability

Not applicable.

## Abbreviation

TSH: Thyroid stimulating hormone

## References

[1] Mori M (2011). Programs for Continuing Medical Education: A session 4 : Clinical guideline for thyroid disease. Nihon Naika Gakkai Zasshi.

[2] Ueshima K (1997). Panic disorder. J Jpn Soc Clin Anesth.

[3] Placidi GPA, Boldrini M, Patronelli A, Fiore E, Chiovato L, Perugi G, Marazziti D (1998). Prevalence of psychiatric disorders in thyroid disease patients. Neruropsychobiology..

[4] Fukao A, Takamatsu J, Arishima T, Tanaka M, Kawai T, Okamoto Y, Miyauchi A, Imagawa A (2020). Graves’ disease and mental disorders. J Clin Transl Endocrinol.

[5] Trzepacz PT, McCue M, Klein I, Levey GS, Greenhouse J (1988). A psychiatric and neuropsychologocal study of patients with untreated Graves’ disease. Gen Hosp Psychiatry.

[6] Stern RA, Robinson B, Thorner AR, Arruda JE, Prohaska ML, Prange AJ (1996). A survey study of neuropsychiatric complaints in patients with Graves’ disease. J Neuropsychiatry Clin Neurosci.

[7] Akamizu T, Satoh T, Isozaki O, Suzuki A, Wakino S, Iburi T, Tsuboi K, Monden T, Kouki T, Otani H, Teramukai S, Uehara R, Nakamura Y, Nagai M, Mori M (2012). Diagnostic criteria, clinical features, and incidence of thyroid storm based on nationwide surveys. Thyroid.

[8] Burch HB, Wartofsky L (1993). Life-threatening thyrotoxicosis: thyroid storm. Endocrinol Metab Clin N Am.

[9] Matsubayashi S, Tamai H, Matsumoto Y, Tamagawa K, Mukuta T, Morita T, Kubo C (1996). Graves’ disease after the onset of panic disorder. Psychother Psychosom.

[10] Ballenger JC, Burrows GD, DuPont RL Jr, Lesser IM, Noyes R Jr, Pecknold JC, Rifkin A, Swinson RP. Alprazolam in panic disorder and agoraphobia: Results from a multicenter trial. Arch Gen Psychiatry. 1988;45(5):413–22. 10.1001/archpsyc.1988.01800290027004 3282478

